# Effectiveness of Interventions to Promote Healthy Eating Habits in Children and Adolescents at Risk of Poverty: Systematic Review and Meta-Analysis

**DOI:** 10.3390/nu12061891

**Published:** 2020-06-25

**Authors:** Rosario Pastor, Josep A. Tur

**Affiliations:** 1 Research Group on Community Nutrition and Oxidative Stress, University of the Balearic Islands, E-07122 Palma de Mallorca, Spain; rosario.pastor@ucavila.es; 2 Faculty of Health Sciences, Catholic University of Avila, 05005 Avila, Spain; 3 CIBEROBN (Physiopathology of Obesity and Nutrition), Instituto de Salud Carlos III, 28029 Madrid, Spain; 4 Foundation Health Research Institute Balearic Islands (IDISBA), University Hospital Son Espases, E-07120 Palma de Mallorca, Spain

**Keywords:** healthy eating habits, diet, health promotion, social class, poverty

## Abstract

The objective of this review was to provide an up-to-date review of trials that include behavioral intervention on the eating habits of children and adolescents at risk of poverty, applying meta-analysis to estimate the size of the intervention effect. A systematic literature search was performed in the following databases: MEDLINE via Pubmed and via EBSCOhost, LILACS and IBECS via VHL. The MeSH terms were used: “social class”, “poverty”, “diet”, “health promotion” (PROSPERO ID: 183900). A total of 14 articles were finally included in this systematic review. The primary results of the included studies were meta-analyzed with the RevMan 5.3 program, assuming a random effects model. Analysis of the overall effect showed combined standardized mean differences (SMD) of 0.16 (CI 95%: [0.03, 0.30], I^2^ = 76%). The *p*-value for the Z statistic was 0.02, which reflects accuracy in the effect estimation. When establishing subgroups based on the intervention tracking time points, studies that provided longer-term follow-up data showed a combined SMD of 0.15 (CI 95%: [−0.02, 0.32], I^2^ = 81%]). Small effects of behavioral interventions were found to promote healthy eating habits, but better effects were shown in cases where the invention was followed up in the long term.

## 1. Introduction

At any time of life, food must provide energy, nutrients and bioactive components necessary for maintaining good health. In childhood, in addition to this function, food should promote optimal growth and development, and family meals should be an educational tool for the acquisition of healthy eating habits, which will have an impact on nutritional behavior in the short, the medium, and the long term. In addition, due to increased school hours and declining family meals, the school has become a critical point for healthy lifestyle education [1]. Adolescence is an ideal time to strengthen these healthy lifestyles, influence educational programs, and avoid risk factors that will remain in the future.

Socioeconomic inequalities in health in terms of morbidity and mortality have been widely described in the literature. People from disadvantaged social backgrounds die younger and suffer disabilities for a greater part of their lives [2,3]. A low socioeconomic position is associated with less healthy dietary patterns, increasing the risk of mortality [4,5,6,7,8,9]. These socio-economic disparities in the diet also involve children and adolescents. Thus, studies showed that the intake of fruits, vegetables, and dairy is higher when the socioeconomic status of the parents is more favorable, while the consumption of sugary drinks and sugary or salty foods with high energy density is higher when socioeconomic conditions are lower [10,11,12,13,14]. However, although it is generally accepted that a low socioeconomic level correlates with lower diet quality, the data come from studies that used different parameters to define poverty and applied different methods for assessing and defining the quality of the diet [15].

Experts agree that behavioral changes provide significant improvements in public health. It has been suggested that interventions changing behaviors showed poor results in low-income populations. Approximately half of interventions targeting people at risk of poverty were effective [16,17,18,19]. A review on the outcome of interventions that promote healthy eating and their impact on social inequalities in the diet [20] found seven studies conducted in seven countries that met the inclusion criteria. Of these seven studies, four targeted only disadvantaged populations, and three targeted the entire population. In three of the four interventions aimed at disadvantaged populations, the results showed an improvement in the diet with increased consumption of fruits and vegetables in two studies, while in the third study, there was an increase in intake of wholemeal bread and skimmed milk. A review that assessed the effectiveness of behavioral interventions targeting diet, physical activity, or smoking in low-income adults showed small effects of interventions on behavior [21]. Another review summarized the effectiveness of obesity prevention and treatment for adolescents in socioeconomically disadvantaged backgrounds using BMI or Z-score BMI as the primary result measure. Relevant secondary results (other adiposity measures such as waist circumference, sagittal abdominal diameter, waist-to-hip ratio, and body fat (%), physical activity, diet, sedentary behavior, and screen time) were also extracted. Five of six studies (four preventive, one treatment studies) measuring dietary behavior reported significant intervention effects. Thus, among these five studies, one reported a significant decrease in consumption of fizzy drinks and foods with high energy density and an increase in fruit and vegetable consumption, two reported a decrease in the intake of sugary drinks, one reported a decrease in energy intake, and another found a decrease in snack and dessert intake. This review found no clear evidence for which strategies were particularly successful giving beneficial effects on BMI in this target group. However, involvement of parents appeared to contribute to the success of weight-related health interventions, and adolescents preferred experiential than didactic activities, mainly for increasing physical activity [22].

None of the reviews described above measured dietary changes as a main outcome, and only one [21] did a meta-analysis to estimate the overall effect of interventions. The main differences of this meta-analysis were the age range over which the interventions were performed, since these were aimed at adults, and that it did not focus on the diet but also measured other parameters, as indicated above. Regarding diet, the results of meta-analysis showed positive effects of interventions with respect to controls. Subgroup analysis showed a maintenance of these small effects of intervention in those studies that provided longer-term follow-up data.

Currently, there is a lack of evidence in effectiveness of interventions on healthy eating habit promotion aimed at children and adolescents at risk of poverty.

The aim of this systematic review is to provide an up-to-date review of trials that included behavioral intervention on the eating habits of children and adolescents at risk of poverty, applying meta-analysis to estimate the size of the intervention effect.

## 2. Materials and Methods

The Preferred Reporting Items for Systematic Reviews and Meta-Analyses (PRISMA) guidance was followed, and the protocol of this systematic review was registered in the international prospective register of systematic reviews (PROSPEROID:183900). A systematic literature search was performed (March 2010–March 2020) in the following databases: Medlars Online International Literature (MEDLINE) via Pubmed and via EBSCOhost, LILACS and IBECS via Virtual Health Library (VHL). The following Medical Subject Headings (MeSH) terms were used: “social class”, “poverty”, “diet”, “health promotion”. The following search strategies were used with Boolean markers: (“Social Class” [MeSH] or “Poverty” [MeSH]) and “Diet” [MeSH]) and “Health Promotion” [MeSH]; and (“Poverty” [MeSH] or “low-income” or “low socioeconomic” or “disavantaged”) and “Diet” [MeSH]) and “Health Promotion” [MeSH]. The search was standardized and carried out by two independent reviewers who proceeded initially to read the titles, then the summaries, and finally the complete article. The articles with disagreement were resolved by consensus. The search was complemented by a review of the reference lists reflected in the articles found to find any original article and obtain additional information.

**Figure 1:**
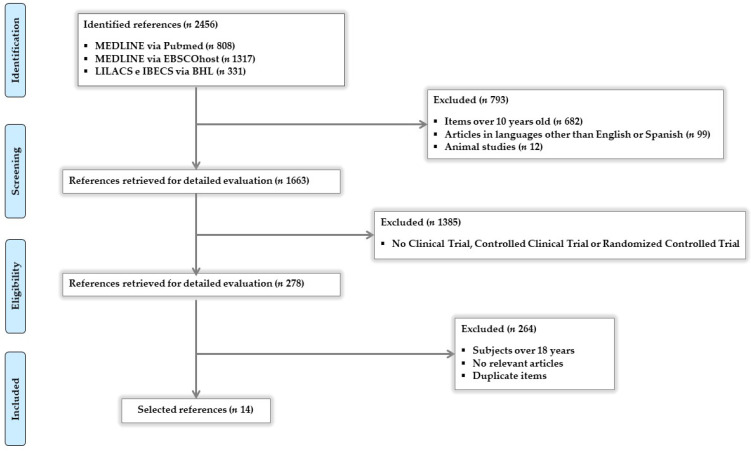
Flow diagram of systematic review.

The process used to identify and select articles is shown in Figure 1. In total, 2456 articles were identified with the above-detailed search criteria. The following search filters were applied: items over 10 years old, articles in languages other than English or Spanish, animal studies (excluded, *n* = 793), trials considered as no clinical trial (CT), controlled clinical trial (CCT), or randomized controlled trial (RCT) (excluded *n* = 1385), subjects over 18 years, no relevant articles, or duplicate items (excluded *n* = 264). A total of 14 articles were finally included in this systematic review. No limitations were included in the search for the country where the study was conducted. For each of the included studies, the data of interest for the purpose of this review were extracted. Data extraction and risk of bias in individual studies were assessed based on standard criteria [23,24]. The publication bias was evaluated using the Begg test as well as its graphic representation (funnel plot) and Egger test.

The data were extracted for all time points reported in each of the studies included. The primary result that was analyzed was the change in eating habits after intervention. For studies where the results were not shown as “overall diet quality” as well as for those who provided follow-up data at different points after the intervention, the combined mean and standard deviations grouped by food groups were analyzed.

The primary results of the included studies were meta-analyzed with the Review Manager (RevMan) version 5.3 program (Cochrane Training, London, UK), assuming a random effects model. As the result variable of the studies was continuous, the means and the standard deviations in the post-intervention of each group (intervention group and control group) were extracted. The standardized mean difference (SMD) was used as an index of the effect of intervention, as studies used different scales to measure the result variable. As the standardized mean difference method does not correct differences in the direction of the measurement scale, in order to ensure that all scales pointed in the same direction for results where a reduction meant a healthy change in eating habits (e.g., reduction of consumption of foods high in saturated fats), the average values of these results were multiplied by −1 before they were introduced into the meta-analysis program. The effect of the intervention for each study was evaluated with its confidence interval (95%), the mean effect (weighting by the inverse of the variance and assuming a random-effects model) with its confidence interval (95%), and its statistical significance test (*p* < 0.05). Heterogeneity statistics (Chi^2^ and I^2^) were calculated, considering the ranges of I^2^ values collected in the Cochrane Handbook for Systematic Reviews of Interventions: 0–40% (non-significant heterogeneity); 30–60%: moderate heterogeneity; 50–90% (significant heterogeneity); 75–100% (considerable heterogeneity) [23].

A subgroup analysis was performed, assuming a random effects model, in order to check whether a moderating variable (i.e., post-intervention follow-up time points) was affecting the size of the effect and could therefore be a factor that partly explained the possible heterogeneity between the results of the studies. The average effect was calculated for each subgroup, its confidence interval, its statistical significance, and its heterogeneity statistics. The Chi^2^ test was also applied to compare the average effects of the subgroups to see if they differed significantly from each other.

## 3. Results

### 3.1. Study Characteristics

#### 3.1.1. Design, Duration, Follow-Up Time, Participants, and Primary Results

Table 1 shows study design, duration and time points, characteristics of the participants and their identification as a population group at risk of poverty, as well as the primary result analyzed.

To initially identify participants as a “low-income population,” the studies were based mainly on the following criteria: place of residence and/or school located in a low-income area [25,26,27,28,29,30,31,32,33,34]; belong to a minority ethnic group identified as a group suffering from income inequality [34,35,36]; enrolled in a financial support program [26,28,31,37,38]. No study specified the level of income that determines the poverty line. However, two studies [25,27] referred to socioeconomic status indexes used in the area where the study was conducted. Most of the studies (*n* = 9) were RCT [25,26,27,29,30,32,35,36,37], three were CCT [31,34,38], and two were CT [28,33].

All studies performed the same measures before and after the intervention for all study groups (intervention and control). However, they differed in the time between the end of the intervention and the most immediate evaluation as well as the number of post-intervention time points. Four studies carried out the measurements at two points after the intervention [26,29,31,33], three in three points of time [25,30,38], five at a single point [27,28,32,35,37], and only one conducted the evaluation at six post-intervention time points [34].

Individual studies included between 50 and 1176 participants, obtaining a total of 6836 participants in the fourteen assessed studies. Three studies focused only on women [25,27,30], and none of them studied only men. Eight studies were aimed at children (6–12 years) [26,28,31,32,34,36,37,38], three at adolescent populations (13 to 18 years) [25,27,35], and three at both population groups [29,30,33]. Only one study included the assessment of dietary intake in secondary outcomes [25]; the rest of the studies included it in the primary outcomes.

#### 3.1.2. Types of Intervention and Methods of Measuring Results

The content of interventions and methods of measuring diet outcomes are described in Table 2. The type of intervention ranged from the provision of educational materials to individual or group advice, in most cases mixing both strategies and establishing multi-component interventions. Six studies targeted both children and their parents/guardians [27,29,31,32,36,38]. The control group received either a dimmed intervention [27,30,31,33,34,35,38] or received no intervention and only participated in data collection during the duration of the study [25,26,28,29,32,36,37]. Within those studies in which the control group received no intervention, some specified that they were offered such intervention at the end of the study [29,36,37].

Regarding the tools used to evaluate the diet, all studies carried out the measurements in all groups (intervention and control) and at all time points. Four studies used a Food Frequency Questionnaire (FFQ) [25,27,32,37], three used a 24 h dietary recall (R24H) [30,35,38], three used questionnaires that included other health measures in addition to dietary intake [28,31,34], three used a specific questionnaire on food and nutrition [29,33,36] among which just one used a Food Frequency Questionnaire along with the specific questionnaire. Finally, a study used observation systems for the four behaviors/environments studied [26].

### 3.2. Risk of Study Bias

The risk of bias for each of the studies included in this review is detailed in Supplementary Table S1. Most studies neither described the protocol of blinding the random assignment nor did they mention the blinding of either party. Additionally, most provided the number of dropouts but not the reason. Finally, only one study stated that it performed an intent-to-treat analysis.

The Begg test (Z = 0.9455; *p* = 0.3444) and the Egger test (t = 0.3124; *p* = 0.7594) show that there is no statistical evidence of publication bias (Figure S1).

### 3.3. Effectiveness of Interventions

#### 3.3.1. Results of Individual Studies and Reported Effects

Table 3 shows the results of diet interventions, and Table 4 summarizes the baseline characteristics and changes in the intervention group with respect to control at different post-intervention time points with respect to the baseline.

Only four studies [25,31,33,37] reported whether there were significant differences in dietary intake in the baseline. Of these studies, two [25,37] found significant differences between the groups, but only one of them [25] reported that this may have been a limitation in the study’s findings. The other studies [31,33] reported significant differences only in intake of some food groups, but neither of the two analyses included what could have caused these differences in the results.

Moreover, one study did not report baseline dietary intake data [29], and another did not report this data either at the baseline or at the post-intervention time point [32]. Finally, one study [28] performed the basal measurements after it applied one of the components of the intervention; therefore, the baseline data were not usable as pretest measures.

#### 3.3.2. Global Effectiveness of Interventions: Data Quantitative Synthesis

**Figure 2:**
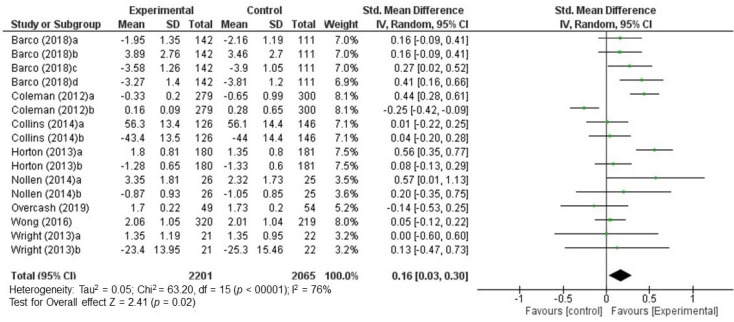
Standardized mean differences (SMD) combined for all post-intervention time points (random effects model).

Among the fourteen studies selected, six could not be used for meta-analysis because data were missing and the original database could not be accessed. The other eight dietary interventions included in the meta-analysis were found to have combined standardized mean differences (SMD) of 0.16 (CI 95%: [0.03, 0.30], I^2^ = 76%) (Figure 2). According to the criteria set out in the Cochrane Handbook for Systematic Reviews of Interventions [23], SMD value showed a small effect of the intervention, reflecting the extent of the confidence interval that, although with some uncertainty, there is precision on the assessment of the size of the effect. The *p*-value for the Z statistic was 0.02, which reflects accuracy in the effect estimation.

In the forest plot, while multiple studies showed mixed effects of the intervention (crossing zero), the overall effect significantly favored a positive effect of intervention on eating behavior.

Sensitivity analyses were carried out through an evaluation of the results by removing the two studies that added less weight to the overall result with the aim of verifying whether or not this caused significant changes in the overall result. The result of this analysis showed only a change in the effect size but not in the effect direction. The results can therefore be considered consistent.

**Figure 3:**
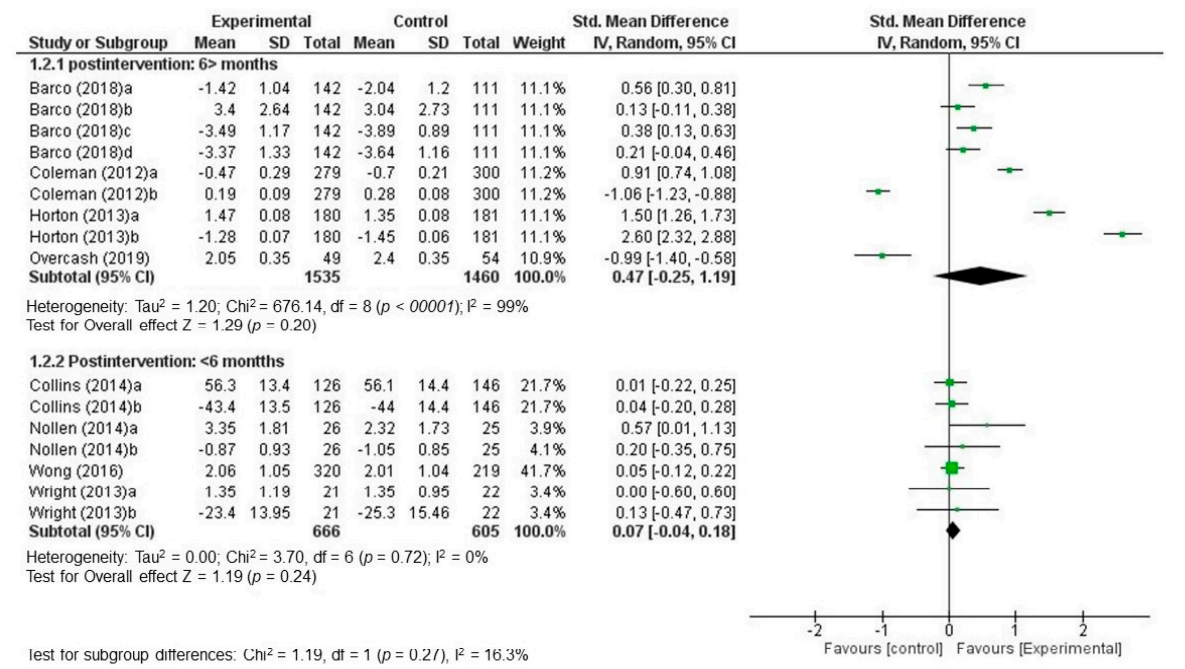
Standardized mean differences combined by subgroups based on post-intervention time points (random effects model).

In establishing subgroups based on intervention follow-up time points, it was found that four studies provided longer-term follow-up data for 6–24 months with a combined SMD of 0.15 (CI 95%: [−0.02, 0.32], I^2^ = 81%]). The *p*-value for the Z statistic was 0.09, which reflected low accuracy in estimating the effect. The Chi^2^ test of comparison between the average effects of subgroups reflected that there were no significant differences *(p* = 0.44) (Figure 3).

**Figure 4:**
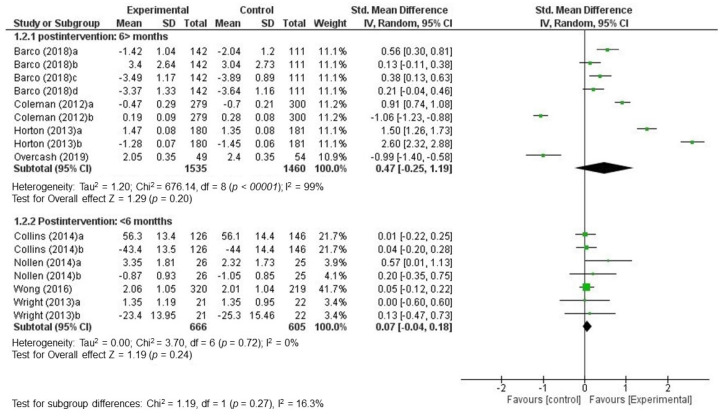
Standardized mean differences combined by subgroups based on the first post-intervention time point (random effects model).

The analysis by subgroups at the first post-intervention time point showed, for studies that analyzed the effects for the first time at 6 months or more post-intervention, a combined SMD of 0.47 (CI 95%: [−0.25, 1.19], I^2^: 99%). The *p*-value for the Z statistic was 0.20, which reflected low accuracy in estimating the effect. The Chi^2^ test of comparison between the average effects of subgroups reflected that there were no significant differences (*p* = 0.27) (Figure 4).

## 4. Discussion

This systematic review provided an up-to-date review of trials (clinical trial, controlled clinical trial, and randomized controlled trial) that included behavioral intervention on the eating habits of children and adolescents at risk of poverty and also applied meta-analysis to estimate the size of the intervention effect. The most important finding of this review was that small effects of interventions were found on eating habits, finding that the overall effect significantly favors a positive effect of intervention on eating behavior. Subgroup analyses suggest better eating behavior outcomes when long-term interventions were monitored (*p*-value for statistic Z = 0.09, for the subgroup that tracked 6 months or more) but without statistically significant differences between the subgroups (*p*-value for Chi^2^ test = 0.04).

Systematic reviews that did not focus on low-income groups reported greater effectiveness of nutritional interventions on the quality of the diet. Thus, in a review evaluating nutritional interventions by Information and Communications Technologies (ICT) on adolescents from high-income countries [39], the authors found that most interventions reported being effective in promoting nutrition-related health benefits. These results match those reported in previous reviews targeting children and adolescents not focused on low-income groups [40,41]. However, in a systematic review aimed to assess the impact of family and preschool/school nutrition programs on the health of children aged 12 and younger in high-income countries [42], the authors reported that school and community nutrition programs in disadvantaged communities were as effective as interventions in other settings.

In general, nutrition programs targeting family and school environments have scarce impact on dietary intake, thus they need to be supported by other useful strategies to create healthy environments, such as increasing the availability of healthy food options and restricting junk food advertising. Another factor that may influence the low effectiveness of interventions is that none of the individual studies implemented long-term nutrition programs. However, large-scale nutrition programs are easier to implement and maintain when funds and supports are available. This was demonstrated in school studies in which nutrition programs were implemented for an extended period [43,44,45]. This is a challenge for future research, as is the impact of the completeness of interventions.

When the meta-analysis was carried out, the results obtained showed consistency in the direction of the effect in favor of the intervention. However, in the analysis carried out at the different time points at which the intervention was tracked, i.e., where the measures resulting from the intervention were performed, statistic I^2^ showed a significant heterogeneity between the studies (I^2^ = 76%). This heterogeneity was explored by performing subgroups, finding that this was higher in the case of studies that followed post-intervention of 6 months or more (I^2^ = 81%), while those who tracked less than 6 months post-intervention showed no heterogeneity (I^2^ = 0). This fact was repeated when the analysis of the effect of the intervention was performed with the same subgroups for the first post-intervention time point. Therefore, meta-analyses confirmed low accuracy of the individual results as well as the small effect on the effectiveness of behavioral interventions on the eating habits of children and adolescents at risk of poverty; however, subgroup analyses suggested better eating behavior outcomes when long-term interventions were monitored. This may be due to the increased involvement of subjects in maintaining these positive dietary changes when they know that they will be monitoring these changes within a certain timeframe, that is, with this follow-up, they would receive a greater encouragement for the maintenance of acquired habits. In addition, in the studies that measured the results of the long-term intervention, during these measures, the professional was able to interact with the measured subject, thus introducing a reinforcement for the maintenance of the acquired behavior.

In the meta-analysis performed by Bull et al. [21], in adults with low socioeconomic status, similar results were reported in this regard with a maintenance of the positive effects on diet in those studies that reported follow-up data. Since no meta-analyses have been performed measuring changes in eating habits after behavioral intervention in children and adolescents, no appropriate comparisons can be done.

Of the fourteen studies included in this review, five involved families. Four of these studies reported statistically significant improvements but only in one of the total parameters studied. Therefore, although parental/family involvement was not a key element in improving children’s eating habits, our findings are in line with the results obtained by Hingle et al. [46] in a 2010 review. The authors concluded that there was insufficient evidence of the impact of parental involvement in dietary interventions aimed at improving children’s eating habits. In contrast, a review by Kader et al. [47] in 2015 confirmed that children’s eating habits can be improved through nutrition education programmed aimed at parents, although the authors show that, in groups with low socioeconomic status, there is a high dropout rate. To implement an effective intervention that involves parents, it is necessary for them to know how to influence their children in terms of ability and opportunity to choose healthy foods [48,49].

This review found a lack of uniformity in the method of measuring child food intake. Reviewed studies included commonly used methods to evaluate dietary intake (24 h recalls, Food Frequency Questionnaire, health questionnaires that include questions about dietary intake, and observation from research staff). However, the variation in the way the method is applied and the absence of uniformity in the food groups assessed resulted in measures different from dietary outcomes. Therefore, uniform approaches need to be developed to assess dietary intake. This can be achieved by paying more attention to the design problems of randomized trials, as Stevens et al. [50] showed in its analysis of the methodological problems of obesity prevention trials.

## 5. Strengths and Limitations

The main strength of this study was that it provided an up-to-date review of trials with behavioral intervention on the eating habits of children and adolescents (12 years old and younger) at risk of poverty, and it also applied meta-analysis to estimate the size of the intervention effects.

In 2018, the United Nations Development Program (UNDP) and the Oxford Poverty and Human Development Initiative (OPHI) developed a new version of the Global Multidimensional Poverty Index (MPI), which complements traditional income-based poverty measures with the deprivations that the individual faces at the same time regarding education, health, and living standards. Our review identified studies that used a wide range of concepts to focus on the low socioeconomic level, such as place of residence and/or school located in a low-income area, belonging to certain ethnic groups, being enrolled in a financial support program, as well as concepts directly linked to low income. In addition, the use of the term “low income” allowed us to implement a clear and objective inclusion criterion for selecting the studies of this review while giving us the possibility to find studies that considered the socioeconomic level low in several ways.

The main limitation of this review was that only eight studies of the 14 that met the inclusion criteria could be included in the meta-analysis due to lack of data, and the original database of these studies could not be accessed. According to Borenstein [51], if we use a fixed effects model, it makes sense to perform a meta-analysis as soon as we have two studies, since a summary effect based on few studies implies a more accurate estimate than the effect reported by each of the individual studies. However, this model assumes that the dispersion of the observed effects is due only to the sampling error. The random effects model assumes that the dispersion of effects is real, at least in part. When this model is based on a small number of studies, the estimate of the variance between studies (T^2^) may be erroneous, because the standard error of the summary effect is based, in part, on this value; therefore, if we present a summary effect with confidence interval, it can provide a false sense of security. However, it is important to note that the direction of the results favored, in a moderate way, the intervention in each of the three analyses carried out. It is necessary to dig deeper into this research based on a major sample size.

Another limitation was the validity and the reliability of dietary consumption data from surveys. Although studies used validated methods such as 24 h recall and Food Frequency Questionnaire, the potential for bias is well known [52,53]. In studies aimed at children, this became more relevant, as they have a greater difficulty in estimating the amount of consumed food and, in the case of surveys being conducted on parents, they have a limited ability to assess all eaten foods outside the family environment. There is evidence that the combined use of various methods of evaluating dietary intake (quantitative and non-quantitative questionnaires) with biomarkers integrated with statistical models can give more accurate estimates of the usual individual intake [54,55]. In addition, no additional strategies were used in our search to locate unpublished revisions (grey literature).

Finally, the studies did not blind random allocation to facilitators or evaluators of results, although blinding behavioral interventions is difficult [56].

## 6. Conclusions

Small effects of interventions were found on food habits, but the overall effect significantly favors a positive effect of intervention in eating behavior. Further research is needed with more effective and long-term interventions to assess what sort of intervention would be the most useful to improve healthy eating habits in children and adolescents of low-income families.

## Figures and Tables

**Table 1:** Description of the reviewed studies: study design, participants.

Study ID [reference] Clinical Trial Identifier, City (Country)	Study Design, Duration and Time Points	Participants	Primary Measure of Result	Identification of the Population at Risk of Poverty
Alaimo (2013) [37] MI (USA)	Randomized Controlled Trial 2 years. Baseline and next school year.	1176 children (55 schools) 12.3 (0.6) years (Mean (SD)).	Nutrient density and healthy food intake	Low income middle schools. Inclusion criteria: having 50% or more of students eligible for free or reduced-price meals.
Barco (2018) [25] NCT02228447 São Paulo (Brazil)	Randomized Controlled Trial. 1 year Baseline, Immediate post-intervention and 6-month post-intervention.	253 girls (10 schools). 14–18 years.	Height and weight, waist circumference. (Dietary behavior is included as secondary outcome, among others).	Public high schools located in low-income areas with medium HDI (There is not low HDI in São Paulo).
Coleman (2012) [26]	Randomized Controlled Trial. 2 years Baseline, first- and second-year post-intervention.	579 children (8 schools). 8.9 (1.6) years (Mean (SD)).	Amount of outside foods and beverages on campuses.	Targeted low-income school district. All children in the district were eligible for free and reduced school meals.
Collins (2014) [27] Hunter, Newcastle and Central Coast regions of New South Wales (Australia)	Randomized Controlled Trial. 1 year. Baseline and 12-month follow up	357 girls (12 schools) 13.18 (0.45) years (Mean (SD))	Percentage energy contributed from nutrient-dense core foods groups and EDNP foods groups.	12 government secondary schools located in the bottom 50% of the SEIFA measure of relative disadvantage.
Evans (2012) [28]	Clinical Trial 4 months. Baseline and post-intervention.	214 children (5 schools). Sixth and seventh-grade students	Dietary intake: fruit and vegetable.	Students who were eligible for free/reduced lunch and breakdown of racial/ethnic minority students. All five schools served students who liven underserved, ethnically diverse communities.
Horton (2013) [29] Imperial County, CA, (USA)	Randomized Controlled Trial. 2 years. Baseline, postintervention, 6-month follow-up (the latter is not described in this paper).	361 mother-child 7–13 years	Dietary intake: fruit and vegetable, sugar-sweetened beverages, fast food. Weight, height, BMI.	Imperial County (CA): is characterized by nationally high poverty rates.
Nollen (2014) [30]	Randomized Controlled Trial. 1 year. Baseline, 4-week follow-up, 8-week follow-up and, 12-week follow-up.	51 girls. 9–14 years.	Dietary intake (fruits/vegetables, sugar-sweetened beverages), scree time, BMI.	Economically disadvantaged neighborhoods.
Nyberg (2016) [31] Stockholm County (Sweden)	Controlled Clinical Trial 6 months. Baseline, post-intervention, 5 months follow-up.	378 children (13 schools, 31 pre-school classes allocated to intervention group or control group 6 years.	Physical activity, dietary intake, screen time, body weight, height and BMI standard deviation.	Three areas in Stockholm County with low employment and low educational level and targeted specifically by the government to support socio-economic development.
Overcash (2019) [38] NCT03641521Minneapolis/St. Paul, MI/WI metropolitan area (USA)	Controlled Clinical Trial. 1 year and 7 months Baseline, immediate post-treatment, 6-month follow-up, 12-month follow-up.	103 parent-child pairs enroll into 1 of 15 location sites. Parent/child (9–12 years).	Total vegetable intake, diet quality (HEI scores), total energy intake, vegetable liking, variety of vegetables tried, child BMI-z score, and home availability of vegetables	Sites serving low-income families including subsidized housing, schools, churches, and community centers. Inclusion criteria: the family must qualify for some form of public assistance.
**Study ID [reference] Clinical Trial Identifier, City (Country)**	**Study Design, Duration and Time Points**	**Participants**	**Primary Measure of Result**	**Identification of the Population at Risk of Poverty**
		- *n* and description		
		- Age		
Rausch (2013) [32] Rosario (Argentina)	Randomized Controlled Trial. 5 months. Baseline, post-intervention.	405 children. 9–11 years (6 schools).	Body weight, height, BMI. Dietary intake.	The school districts that participated in this study comprised neighborhoods of vulnerable social sectors in which macroeconomic conditions are compatible with very-low, low, and lower-middle income standards.
Rees (2010) [35] London and the West Midlands (UK)	Randomized Controlled Cluster Trial. 3 months. Baseline and 3-month follow-up.	823 children (8 schools) 12–16 years.	Dietary intakes: brown bread, wholegrain cereal, fruit and vegetables.	The areas were contacted based on their representation of ethnic minority and low-income groups.
Tamiru (2016) [33] Jimma Zone, Oromia Regional State, (Ethiopia)	Clinical Trial. 9 months. Baseline, midline (6th month) and end line (9th month).	992 Mother-Child pairs (4 pairs primary schools). 13.6(1.99) years (Mean (SD))	Quality diet, temporary hunger, body weight, height.	Jimma Zone: total population of 2.5 million; 94% living in the rural settings.
Wong (2016) [34] NCT00994084 Houston, TX, (USA)	Controlled Clinical Trial. 18 weeks Baseline and at the end of each of the 3 6-week sessions.	1094 children (14 community center)). 10.2(0.1) years (Mean (SE)).	Body weight, height, dietary habits, nutrition knowledge, self-esteem, and physical activity.	Community centers located in low-income neighborhoods within the City of Houston. Hispanic o black children.
Wright (2013) [36] Boston, MA, (USA)	Randomized Controlled Trial. 1 year. Baseline and 3 months post intervention.	50 parent-child 9–12 years obese children.	BMI, intakes of calories, fat, fruits and vegetables, television viewing later.	Families from underserved populations. African American (72%).

Abbreviations: EDNP: energy-dense nutrient-poor; HDI: human development index; HEI: healthy eating index; SEIFA: socioeconomic index for areas; SES: socioeconomic status.

**Table 2:** Types of intervention and dietary outcome measure.

Study ID, [Reference]	Intervention Group Description	Control Group Description	Dietary Outcome Measures
Alaimo (2013) [37]	Intervention group: (1) HSAT Only; (2) Student SNAK Team; (3) MSBE Nutrition Policy. Intervention elements: CSHT (1, 2, 3), HSAT and Action Plan (1, 2, 3), Make ≥ 1 nutrition education/marketing change (1, 2, 3), SNAK (2 group), MSBE (3). Learning cycles.	Participated only in data collection during the study period (were offered the intervention post study).	Diet assessment measures from FFQ pre- and postintervention (55 school) in 4 groups of the 2 outcome measurement time points.
Barco (2018) [25]	Multicomponent school-based intervention (6 months).	Participated only in data collection during the study period	BFFQ-FP previously tested for reliability and relative validity in the 2 outcome measurement time points.
Coleman (2012) [26]	Target strategies (classroom, before/after school, recess, cafeteria (school meals)). Do-Study-Act (PDSA)	Participated only in data collection during the study period	Three observation system for the four main nutrition-related organizational behaviors/environments: - School lunch/cafeteria, morning snack recess/playgrounds in elementary: observations based upon what children were consuming. - Classrooms/school-wide events: systematic observation of school trash.
Collins (2014) [27]	Nutrition handbook that included 10 weeks of health information for parents. Three practical nutrition workshops. Newsletters and text messaging. 10-week delivery of health information by teachers in the schools.	Nutrition handbook that included 10 weeks of health information for parents. Three practical nutrition workshops. Newsletters and text messaging.	ACAESFFQ (was previously evaluated for reliability and validity in Australian school students to 9–16 years) in the 2 outcome measurement time points.
Evans (2012) [28]	Intervention (6 component) for a 5-month period (opportunity to participate in any of the six SHK intervention components).	Participated only in data collection during the study period.	Fruit and vegetables consumption (SHK questionnaire), baseline and post-intervention.
Horton (2013) [29]	DVD series and family manual. Home visit (1 visit/week for 2 months, followed by 1 visit/2 weeks during the third month with telephone calls on non-visit weeks, and a final home visit and telephone call during the fourth month): family support for healthy eating and to maximize sustainability of family behavior change.	Participated only in data collection during the study period (received the DVD series and family manual after completing the final assessment protocol)	Daily intake fruit and vegetable: 2 questions from the National Cancer Institute Food Attitudes and Behavior survey. Daily servings of sugar-sweetened beverages intake: 1 question on number of cans or glasses. Weekly fast food intake: asking how many days they ate in a typical week.
Nollen (2014) [30]	Three 4-week modules that targeted fruits/vegetables, sugar-sweetened beverages, scree time. Mobile intervention: real-time goal setting, self-monitoring, tips, feedback, and positive reinforcement.	Three 4-week modules that targeted fruits/vegetables, sugar-sweetened beverages, screen time. Written manual: same content as in mobile intervention but no prompting.	2 standardized 24 h dietary recall in 3 outcome measurement time points.
Nyberg (2016) [31]	Health information for parents (brochure). Two individual sessions of Motivational Interviewing for parents (2 × 45 min). Teacher-led classroom activities children (10 × 30 min).	Health information for parents (brochure). Teacher-led classroom activities children (10 × 30 min).	Validated (against 24 h dietary recall) parent-proxy questionnaire, the EPAQ in the 3 outcome measurement time points.
Overcash (2019) [38]	Cooking Matters program (6 weekly classes). Behavioral Strategies segment: 6 total.	Cooking Matters program (6 weekly classes)	Three 24 h dietary recall immediately following in the 4 outcome measurement time points.
Rausch (2013) [32]	4 workshops: three for the children (Healthy Eating, Body in Motion, and Healthy Body); and one for their parents/caregivers (40 min) Modifications to the school cafeteria menu.	Participated only in data collection during the study period	WFFQ baseline and post-intervention.
Rees (2010) [35]	Received a leaflet tailored to their responses to a baseline diet and psychological questionnaire.	Received a copy of a comparable generic leaflet based on national guidelines, which was not tailored.	Three 24 h dietary recall sheets over three different days at baseline, and again at follow-up 3 months later,
Tamiru (2016) [33]	School-based health and nutrition education. Enabling school-health environment.	Enabling school-health environment.	FANTA individual dietary-diversity questionnaire and 1 food-frequency at baseline and the 2 outcome measurement time points.
Wong (2016) [34]	30 min of nutrition or healthy habits lessons twice a week (3–6-week sessions).	Took part in regular after-school childcare enrichment programs at community centers. Twice a week: Science is Fun activities.	A multiple-choice quiz in the 3 outcome measurement time points.
Wright (2013) [36]	12 weeks telephone counseling intervention delivered by automated IVR system and an HER behavioral counseling tool used by the primary care clinician during follow- up visits (parents and children: similar but separate interventions).	Participated only in data collection during the study period (were offered the intervention post study).	Children: Block Dietary Data System Kids Food screener. Parents: Block 2007 screener Fat, Sugar, Fruit and Vegetable screener. In the 3 outcome measurement time points.

Abbreviations: ACAES: Australian Child and Adolescent Eating Survey; BFFQ-FP: Brazilian Food Frequency Questionnaire based on the food pyramid; CSHT: Coordinated School Health Teams; EPAQ: Eating Physical Activity Questionnaire; FANTA: Food and Nutrition Technical Assistance; FFQ: Food Frequency Questionnaire; HSAT: Healthy School Action Tools; IVR: Interactive Voice response; MSB: Michigan State Board of Education; SHK: Sprouting Healthy Kids; SNAK: School Nutrition Advances Kids; WFFQ: weekly FFQ.

**Table 3:** Intervention results.

Study ID [Reference]	Outcome Measure	Baseline N/M (SD or SE)				Follow-Up N/M (SD or SE)			
		Intervention Group		Control Group		Intervention Group		Control Group	
Alaimo (2013)a [37]	Total vegetables (cup/day).	134 ^ɤ^	1.20 (0.95) *	228 ^ɤ^	1.10 (0.94) *	ND	ND	ND	ND
Alaimo (2013)b [37]	% kcal from saturated fat and total fat.	ND	20.03 (4.87) *	228 ^ɤ^	21.69 (4.11) *	ND	ND	ND	ND
Barco (2018)a [25]	Animal food group: milk, meats (servings/day).	142	2.13 (1.40) *	111	2.12 (1.05) *	142	1.95 (1.35) *	111	2.16 (1.19) *
Barco (2018 )b [25]	Vegetarian food group: rice, veggies, fruits, beans(servings/day).	142	3.77 (2.75) *	111	3.77 (2.48) *	142/	3.89 (2.76) *	111	3.46 (2.70) *
Barco (2018)c [25]	Oils group (servings/day).	142	3.97 (1.22)	111	3.95 (1.38)	142	3.58 (1.26) *	111	3.90 (1.05) *
Barco (2018)d [25]	Sweets group (serving/day).	142	4.33 (1.13)	111	4.30 (1.14)	142	3.27 (1.40) *	111	3.81 (1.20) *
Coleman (2012)a [26]	Outside unhealthy foods and beverages (items/child/week).	279	0.37 (0.17) *	300	0.51 (0.21) *	279	0.33 (0.20) *	300	0.65 (0.99) *
Coleman (2012)b [26]	Outside healthy foods and beverages (items/child/week).	279	0.15 (0.09) *	300	0.19 (0.07) *	279	0.16 (0.09) *	300	0.28 (0.65) *
Collins (2014)a [27]	Energy from core foods (%).	158	54.8 (13.5)	172	55.4 (13.8)	126	56.3(13.4)	146	56.1 (14.4)
Collins (2014)b [27]	Energy from no-core foods (%).	158	45.2 (13.5)	172	45.0 (1.0)	126	43.4 (13.5)	146	44.0 (14.4)
Evans (2012) [28]	Vegetables and fruits (servings/day)	ND	ND	ND	ND	ND	ND	ND	ND
Horton (2013)a [29]	Vegetables and fruits (cup/day).	ND	ND	ND	ND	180	1.47 (0.81) *	181	1.35 (0.80) *
Horton (2013)b [29]	Sugar-sweetened (servings/day) and fast food (days/week).	ND	ND	ND	ND	180/	1.28 (0.65) *	181	1.33 (0.60) *
Nollen (2014)a [30]	Fruits and vegetables (servings/day).	26	2.53 (1.45)	25	2.34 (1.55)	26	3.35 (1.81)	25	2.32 (1.73)
Nollen (2014)b [30]	Sugar-sweetened beverages (servings/day).	26	1.20 (0.92)	25	0.95 (0.87)	26	0.87 (0.93)	25	1.05 (0.85)
Nyberg (2016)a [31]	vegetables, fruits (servings/day).	185	0.72 (0.61) *	193	0.83 (0.69) *	ND	ND	ND	ND
Nyberg (2016)b [31]	Fruit juice, milk flavored, soft drinks, snacks, chocolate/sweets, ice-cream, cake/buns/cookies	185	0.37 (0.57) *	193	0.50 (0.70) *	ND	ND	ND	ND
Overcash (2019) [38]	Total vegetable servings	49	1.7 (0.2)	54	1.6 (0.2)	49	1.7 (0.22) *	54	1.73 (0.2) *
Rausch (2013) [32]	Vegetables, fruits, Skim milk, cereals, juice (servings/week).	ND	ND	ND	ND	ND	ND	ND	ND
Rees (2010) [35]	Brown bread, wholegrain cereal, fruits and vegetables (servings/day).	406	0.56 (0.71) *	417	0.52 (0.67) *	ND	ND	ND	ND
Tamiru (2016) [33]	Consumption of animal source	ND	ND	ND	ND	ND	ND	ND	ND
Wong (2016) [34]	Fruits and vegetable	524	2.13 (0.04)	353	2.15 (0.04)	320 ^ɤ^	2.06 (1.05) *	219 ^ɤ^	2.01 (1.04) *
Wright (2013)a [36]	Vegetables and fruits (cup/day)	21	1.15 (0.86) *	22	1.35 (0.95) *	21	1.35 (1.19) *	22	1.35 (0.95) *
Wright (2013)b [36]	Saturated Fat and Total Fat (g/day)	21	25.75 (16.49) *	22	26.20 (16.31) *	21	23.40 (13.95) *	22	25.30 (15.46) *

Abbreviations: ND: unavailable “N/M(SD or SE)”. * Combined means (combined standard deviation or combined standard error). ^ɤ^ Average sample size of the different points of follow-up.

**Table 4:** Baseline characteristics and changes between baseline and follow-up.

Study ID [Reference]	Baseline Characteristics	Changes between Baseline and Follow-Up for the Intervention Group, with Respect to the Control Group
Alaimo (2013) [37]	School characteristics at baseline: no significant differences among groups. Student dietary intake: significant differences at baseline and all subsequent analyses adjusted for baseline dietary values. The authors showed the post-intervention results adjusted for baseline value, interaction of race/gender, kitchen type, urbanization, and percent of children eligible for free/reduced-price meals.	HSAT Only: significantly increase fruit intake (17.3%) and fruit juice (>13.8%). SNAK Team: increased added sugars intake (14.6%). MSBE Policy: increased fruit intake (18.3%).
Barco (2018) [25]	The authors reported dietary intake data in the baseline. Although groups were randomized after baseline assessments, statistical differences between groups at baseline were detected. Showed means (SD), Cohen’s d and *p*-value at baseline, and two post-intervention time points.	No significant changes in dietary intake were found.
Coleman (2012) [26]	The authors reported dietary intake data in the baseline, but not whether there were significant differences among the groups. Showed means (SD), F, *p*-value” at baseline and two post-intervention time points.	Unhealthy and healthy food and beverage items/child/week: outside unhealthy food items on intervention school campuses decreased over time (*p* < 0.001) while these items increased over time in control schools (*p* = 0.02). Unhealthy drink items on intervention school campuses decreased over time (*p* = 0.015) and control schools did not change. Outside healthy food items on intervention school campuses decreased (*p* = 0.03) and control school items did not change over time.
Collins (2014) [27]	The authors reported dietary intake data in the baseline but not whether there were significant differences among the groups. Showed means (SD), time *p*-value and group by time *p*-value at baseline, and post-intervention time point.	Percentage of energy from EDPN foods: >44% in both groups at baseline and this remained high at 12 months. Sweetened beverage/day: reduction in consumption, with a greater increase in the proportion consuming less than one sweetened beverage per day compared to the control girls (24–41% vs. 34–37%, *p* = 0.057).
Evans (2012) [28]	The collection of baseline data took place a few weeks after the “cafeteria” component of the intervention was applied, and therefore the baseline data were not usable as pretest measures. Showed means, estimated difference between treatment and control (SE), and *p*-value according to exposure to one or more/two or more intervention components, controlling for gender, race/ethnicity, and SES.	Since the comparison group includes students with exposure to one or more SHK components, treatment effects are relatively attenuated. Compared with students who were exposed to fewer than two components in SHK intervention, students who were exposed to two or more of the components scored significantly higher on fruit and vegetables intake, self-efficacy, and knowledge measures at posttest (*p* < 0.05) and significantly lower on the preference for unhealthy foods scale (*p* < 0.01).
Horton (2013) [29]	The authors did not report dietary intake data on the baseline. However, they showed the post-intervention results adjusted for baseline value, mother´s race, education, and marital status (means (SE), *p*-value).	Intervention effects were observed on weekly fast food consumption (*p* < 0.05) and 0.14 more daily cups of vegetables, which was not statistically significant.
Nollen [30]	The authors reported dietary intake data in the baseline but not whether there were significant differences among the groups. They reported that the seven girls lost to follow-up (four MT and three control) did not differ from the 44 completers on total energy or percentage of calories obtained from fat. Showed means (SD), Cohen’s d and *p*-value (between group and within group) at baseline, and two post-intervention time point.	Intervention group exhibited trends toward increased FVs (+0.88, *p* = 0.08) and decreased SSBs (−0.33, *p* = 0.09). The adjusted difference between groups of 1.0 servings of FV (*p* = 0.13) and 0.35 servings of SSB (*p* = 0.25) was not statistically significant but indicated small to moderate effects of the intervention.
Nyberg (2016) [31]	The authors reported dietary intake data in the baseline. There were no significant baseline differences between the groups except for intake of ice-cream, chocolate, and sweets, with children in the control group consuming significantly more than those in the intervention group. Post-intervention time point: results of mixed Poisson regression adjusted for sex, parental education and baseline value (regression coefficient beta, *p*-value between intervention and control groups, CI = 95%)	At baseline, 70% of the participating children consumed at least 2 servings of fruit and vegetables daily at home. Forty percent of the children consumed at least one serving of unhealthy foods daily at baseline. At T2, the intervention group had a significantly lower intake of unhealthy foods (aggregated snacks, ice-cream, cookies, and sweets) (*p* (*p* = 0.01). This effect was sustained in boys at T3 (*p* = 0.03). Intake of unhealthy drinks (soft drink, flavored milk, and fruit juice above 1 serving) at T2 was significantly lower in the intervention group (*p* = 0.01) compared to the control group. This effect was not sustained at T3.
Overcash (2019) [38]	The authors reported dietary intake data in the baseline, but not whether there were significant differences among the groups. Sowed LSM (SE), *p*-value at baseline and three post-intervention time points.	Total vegetable intake as well as intake for all but 1 of the individual vegetables measured (legumes at baseline, *p* = 0.04) did not significantly differ between the intervention and control children at any of the 4 time points (baseline and three post-intervention time point).
Raush (2013) [32]	The authors did not provide intakes in either the baseline (T1) or the time point (post-intervention). They compared the percentage of children who showed a positive change versus the negative change in healthy food intake before and after the intervention.	Girls in the experimental group tended to increase their intake of the five foods targeted by the program; this attained statistical significance for skim milk (*p* = 0.03) and orange juice (*p* = 0.05). Girls of the control group showed a decrease (albeit non-significant) in their intake of skim milk and of low-sugar cereals.
Rees (2010) [35]	The authors reported dietary intake data in the baseline but not whether there were significant differences among the groups. Sowed change in dietary intake (number of servings/day) from baseline to follow-up.	Intake of brown bread increased from 0.39 to 0.51 servings/day in the intervention group with a smaller but significant increase in the control group also (from 0.28 to 0.35 servings/day). For the other foods, there were no significant effects of the tailored intervention above that of the control intervention.
Tamiru (2016) [33]	The variety of dietary intake of both the intervention and the control schools at baseline was almost similar except for protein source food and oil consumption. Mean difference (SE), *p*-Value (baseline, midline, and end line)	There was a significant difference (*p* < 0.001) between the intervention and control schools where there was a significant improvement of animal source food consumption among intervention schools (*p* < 0.001).
Wong 2016 [34]	The authors reported dietary intake data in the baseline but not whether there were significant differences among the groups. Sowed means (SE), *p*-value (baseline and the end of the 3–6-week sessions)	The fruit/vegetable scores and the nutrition label score were not different between the 2 groups (*p* ≥ 0.67).
Wright (2013) [36]	The authors reported dietary intake data in the baseline but not whether there were significant differences among the groups. Sowed means (SE), *p*-value (baseline and post-intervention time point).	There were no statistically significant between group differences.

Abbreviations: DE: standard deviation; EDNP: energy-dense nutrient-poor; FVs: fruits and vegetables; LSM: least square means; SSBs: sugar-sweetened beverages; SE: standard error; SHK: Sprouting Healthy Kids.

## Supplementary Materials

The following are available online at https://www.mdpi.com/2072-6643/12/6/1891/s1, Table S1: Risk of bias for individual studies, Figure S1: Funnel plot.
