# Supplementary material for: Effectiveness of Interventions to Promote Healthy Eating Habits in Children and Adolescents at Risk of Poverty: Systematic Review and Meta-Analysis
Source: Nutrients. 2020 Jun 25;12(6):1891. doi: 10.3390/nu12061891 (PMC7353268; doi:10.3390/nu12061891)
Supplement: Supplementary file 1 [file nutrients-12-01891-s001.pdf]

First author and reference	Quality of random allocation concealment	Description of withdrawals and dropouts	Intention to treat analysis?	Participants blinded to treatment status?	Intervention facilitators blinded to treatment status?	Outcome assessors blinded to treatment status?
Alaimo (37)	Bi	No	No	Bi	Bi	Bi
Barco (25)	A	No	No	Ai	Ai	Ai
Coleman (26)	Bi	Numbers	No	Bi	Bi	Bi
Collins (27)	Bi	Numbers and reasons	No	Bi	Bi	Bi
Evans (28)	C	Numbers	No	C	C	C
Horton (29)	Bi	Numbers	No	Bi	Bi	Bi
Nollen (30)	Bi	Numbers	No	Bi	Bi	Bi
Nyberg (31)	Bi	Numbers	No	Bi	Bi	Bi
Overcash (38)	Bi	Numbers and reasons	No	Bi	Bi	Bi
Rausch (32)	Bi	Number	No	Bi	Bi	Bi
Rees (35)	Bi	Numbers	No	Bi	Bi	Bi
Tamiru (33)	C	Numbers	No	C	C	C
Wong (34)	C	Numbers	No	C	C	C
Wright (36)	A	Numbers	Yes	Ai	Ai	Ai

Quality of random allocation concealment: A: good attempt at concealment; Bi: states random allocation but no description given of concealment; Bii: attempt at concealment but real chance of disclosure of assignment prior to formal trial entry; C: not concealed.
Blinding: Ai: action taken at blinding likely to be effective; Aii: blinding stated but no description gives; Bi: no mention of blinding; Bii: attempt at blinding but reason to think it may not have been successful; C: not blinded.
